# The Similarities and Distances of Growth Rates Related to COVID-19 Between Different Countries Based on Spectral Analysis

**DOI:** 10.3389/fpubh.2021.695141

**Published:** 2021-09-23

**Authors:** Ray-Ming Chen

**Affiliations:** School of Mathematics and Statistics, Baise University, Baise, China

**Keywords:** growth rate, COVID-19, Manhattan metric, similarity measures, spectral analysis

## Abstract

The COVID-19 pandemic has taken more than 1.78 million of lives across the globe. Identifying the underlying evolutive patterns between different countries would help us single out the mutated paths and behavior of this virus. I devise an orthonormal basis which would serve as the features to relate the evolution of one country's cases and deaths to others another's via coefficients from the inner product. Then I rank the coefficients measured by the inner product via the featured frequencies. The distances between these ranked vectors are evaluated by Manhattan metric. Afterwards, I associate each country with its nearest neighbor which shares the evolutive pattern via the distance matrix. Our research shows such patterns is are not random at all, i.e., the underlying pattern could be contributed to by some factors. In the end, I perform the typical cosine similarity on the time-series data. The comparison shows our mechanism differs from the typical one, but is also related to each it in some way. These findings reveal the underlying interaction between countries with respect to cases and deaths of COVID-19.

## 1. Introduction

COVID-19 is in full, the COVID-19 pandemic is still ongoing and is spreading across all the continents (1, 2). The spread of this pandemic is has been studied by many researchers (3–6). There are many ways to look into the behaviors of the viruses or the pandemic itself (7, 8) for the sake of efficacy of travel bans or vaccines (9). Some researches have even have established the relations between cases and deaths of COVID-19 from demographic, economic, and social perspectives (10). In this article, I devise an orthonormal basis (11) 𝔹_*N*_ which is motived by Fourier analysis (12) and could thus take the underlying frequencies of data into consideration. I utilize the COVID-19 database (13) which records the weekly COVID-19 cases and deaths from Week 15 to 51 (37 weeks in total). By filtering out some non-essential data (countries), I obtain 90 countries as our research targets. By calculating the 36 (the number of intervals from Week 15 to 51) growth rates of the cases and deaths for the 90 countries, I have an input vector. By transforming this vector into a set of coefficients, which is the results of inner product via 𝔹_36_, I start to rank the coefficients by positive integers,: from 1 to 36. The ranks indicate the strength (relation) between the input vector and the underlying frequencies. A larger coefficient will be assigned a larger positive integer. By doing so, I have a 90 × 36 coefficient matrix, where 90 is the number of the sampled countries and 36 is the number of frequencies (or the length of the input vector). Then, I use Manhattan metric (14) to measure the distances between all the ranked vectors and yield a 90 × 90 distance matrix. Afterwards, I associate each country with its nearest neighbor via the minimal distance in the distance matrix. In the end, I rerun our data with another typical approach: cosine similarity, which could be calculated either from the original time-series data or the transformed frequency coefficients, i.e., both would produce the identical results by the property of an inner product. The interaction between these two approaches are also revealed via Jaccard Index (15). Our research shows that the patterned evolutive correlation between counties not random, i.e., there are some fundamental factors that contribute to such relation. The research also reveals that the correlated patterns for cases and deaths between countries bears no similarity at all. This also indicates that there is a strong discrepancy between evolution of cases and the one of deaths.

## 2. Methodology and Procedures

I devise a class of orthogonal bases, which are serve as our feature extractors. Then a complete set of procedures are is also described in this section.

### 2.1. Orthogonal Basis

Motivated by the Fourier series and Fourier transform, I devise an orthonormal basis which is easier and much more intuitive to adopt and interpret the analysis of, since it involves only the real numbers—not the complex numbers, which normally are harder to use to interpret the analyzed results.

Let ℕ denote the set of positive integers. Suppose $\vec{v}$ is a vector whose elements are all non-negative integers. ${\vec{v}}_{i}$ is used to denote its *i*'th element in the vector and $\left|\vec{v}\right|$ is used to denote its length. Let us assume $|\vec{v}|=N+1$, where *N* stands for a natural number in this article. I use $\Delta \vec{v}$ to denote its growth vector, i.e., $\Delta \vec{v}=\left(\frac{{\vec{v}}_{2}-{\vec{v}}_{1}}{{\vec{v}}_{1}},\frac{{\vec{v}}_{3}-{\vec{v}}_{2}}{{\vec{v}}_{2}},\cdots \;,\frac{{\vec{v}}_{N+1}-{\vec{v}}_{N}}{{\vec{v}}_{N}}\right)$. Observe that $|\Delta \vec{v}|=N$. This growth vector is our main research target, since I study the (weekly) growth rates of cases and deaths regarding COVID-19. Later on, I would tweet tweak the definition of growth vector slightly to fit our analytical purpose. For any two vectors $\vec{v}$ and $\vec{w}$, I use $<\vec{v},\vec{w}>$ to denote their inner product. Define real functions $I\left(x\right)=\sqrt{\frac{1}{N}}$ and $b_{m}\left(x\right):=\sqrt{\frac{2}{N}}\cdot cos\left[\left(\frac{2\pi }{N}\cdot m\right)\cdot x\right]$ and ${\tilde{b}}_{m}\left(x\right):=\sqrt{\frac{2}{N}}\cdot sin\left[\left(\frac{2\pi }{N}\cdot m\right)\cdot x\right]$, where *x* ∈ {1, 2, ⋯ , *N*}. Define


$$\begin{array}{l} {𝔹}_{N}\\ :\;=\{\begin{array}{ll} \left\{I,b_{1},{\tilde{b}}_{1},b_{2},{\tilde{b}}_{2},\cdots ,b_{m},{\tilde{b}}_{m},\cdots ,b_{\frac{N}{2}-1},{\tilde{b}}_{\frac{N}{2}-1},\sqrt{\frac{1}{2}}\cdot b_{\frac{N}{2}}\right\} & \text{, if }N\text{ is even;}\\ \left\{I,b_{1},{\tilde{b}}_{1},b_{2},{\tilde{b}}_{2},\cdots ,b_{m},{\tilde{b}}_{m},\cdots ,b_{\frac{N-1}{2}},{\tilde{b}}_{\frac{N-1}{2}}\right\} & \text{, if }N\text{ is odd.} \end{array} \end{array}$$

By some manipulation of mathematical operations, 𝔹_*N*_ is provend to be an orthogonal basis for all natural number *N*.

### 2.2. Procedures

**Figure 1:**
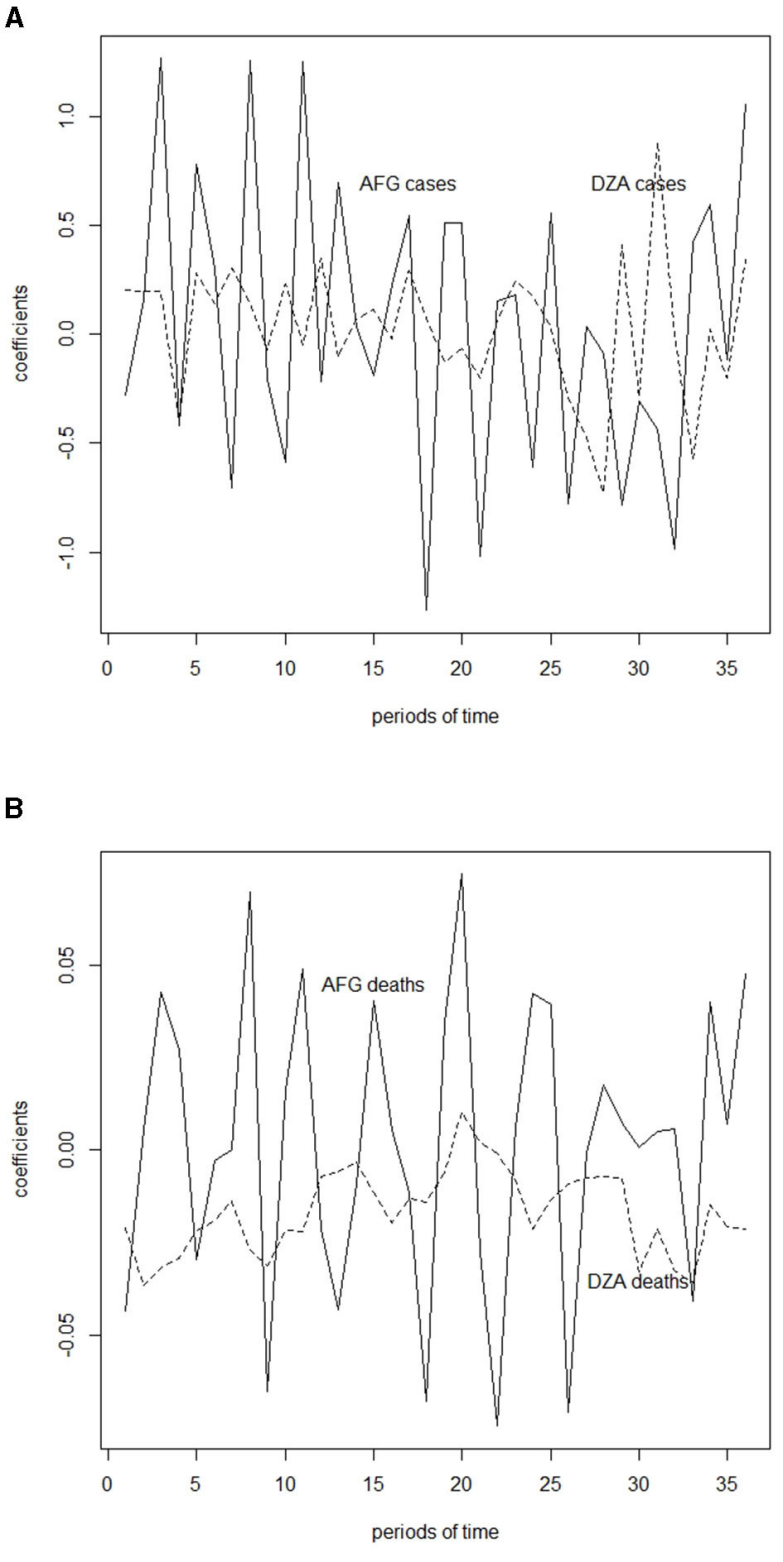
Inner product of case and death growth rates and featured frequencies, which is calculated in Table 1 for Afghanistan (AFG) and Algeria (DZA). **(A)** Inner product of case growth rates and featured frequencies for Afghanistan (AFG) and Algeria (DZA). **(B)** Inner product of death growth rates and featured frequencies for Afghanistan (AFG) and Algeria (DZA).

In this section, I describe a procedure to analyze (in the form of a matrix) *M* × (*N* + 1) time-series data, where *M* is the number of the sets and *N* + 1, which is the number of points of time. The purpose for adding 1 is to simplify our further analysis which utilizes its difference (or *N* intervals). The whole analytical steps go as follows:

1. Specify the *M* researched subjects (for example, countries) and *N* + 1 points of times (for example, weeks). Then collect the sets of time-series data which could then be represented by $\left\{{\vec{v}}^{1},{\vec{v}}^{2},\cdots \;,{\vec{v}}^{M}\right\}$, where each $|{\vec{v}}^{k}|=N+1$.
2. Calculate the growth vector for growth rate of each ${\vec{v}}^{k}$ by $\Delta {\vec{v}}_{i}^{k}=\frac{{\vec{v}}_{i+1}^{k}-{\vec{v}}_{i}^{k}}{1+{\vec{v}}_{i}^{k}}$, where 1 ≤ *i* ≤ *N* for all *k* ∈ {1, 2, ⋯ , *M*}. Here (for our analytical purpose) the denominator is deliberately added by 1 to avoid the divisor being 0.
3. Calculate the inner products or coefficient vector for each growth vector $\Delta {\vec{v}}^{k}$ by $<\Delta {\vec{v}}^{k},\vec{b}>$, where $\vec{b}\in {𝔹}_{N}$ for all *k* ∈ {1, 2, ⋯ , *M*}. Let ${𝔹}_{N}\left(\Delta {\vec{v}}^{k}\right)$ denote the corresponding coefficient vector. These coefficients serve as the extracted features of the growth rates, as shown in Figure 1.
4. Rank ${𝔹}_{N}\left(\Delta {\vec{v}}^{k}\right)$ via positive numbers in which the higher the value is among ${𝔹}_{N}\left(\Delta {\vec{v}}^{k}\right)$, the higher the positive integer assigned is assigned. Let us call this ranked vector $R{𝔹}_{N}\left(\Delta {\vec{v}}^{k}\right)$.
5. Calculate the distances between all the ranked vectors via Manhattan metric *d* among all the *M* subjects that would result in a distance matrix ${\left[d\left(R{𝔹}_{N}\left(\Delta {\vec{v}}^{k}\right),R{𝔹}_{N}\left(\Delta {\vec{v}}^{h}\right)\right)\right]}_{k,h=1}^{M}$.
6. Find the minimal pairs (or nearest neighbors) for all the subjects with least distance via the above distance matrix.

## 3. Results

In correspondence to section 2, I embark on data analysis and produce the results in this section. I download the historical weekly data (up to Week 51, 2020) of the reported COVID-19 cases and deaths worldwide. In order to avoid biased sampling, I filter the data according to the following criteria:

1. Among all the countries, only the populations with of more than 10 millions are included;
2. Only data from Week 15 to 51, Year 2020 are taken as samples.

First of all, the global weekly data regarding COVID-19 are read from its source file (13) and stored in a matrix DT whose size is 9,152 by 10. After filtering out the non-essential samples by the above criteria, I obtain 90 countries (with abbreviated country codes and corresponding labels) as shown in Table 1—each of which contains 37 weekly data (from Week 15 to 51). Furthermore, each country is represented by a 37 by 2 matrix, where 2 indicates the two columns chosen (cases weekly and deaths weekly) out of the original ten columns. An example of such matrices for Country AFG and DZA are listed in Table 2. Data for other countries are omitted here for limited space. In the table, “1c” denotes the cases of COVID-19 in AFG; “2c” denotes the cases of COVID-19 in DZA; “1d” denotes the deaths of COVID-19 in AFG and “2d” denotes the deaths of COVID-19 in DZA. Based on this table, I start to calculate the weekly growth rates for cases and deaths by the formula


$$\Delta_{n}=\frac{Week(n+1)-Week(n)}{1+Week(n)},$$

where *Week*(*n*) denotes the growth rates for cases or deaths at Week *n*. Observe that 1 is added to the denominator to avoid the infinite growth rate. An example of cases and deaths regarding the growth rates for AFG and DZA are presented in Table 3.

**Table 1:** Numbers representing sampled countries.

**1**	**2**	**3**	**4**	**5**	**6**	**7**	**8**	**9**	**10**
AFG	DZA	AGO	ARG	AUS	AZE	BGD	BEL	BEN	BOL
11	12	13	14	15	16	17	18	19	20
BRA	BFA	BDI	KHM	CMR	CAN	TCD	CHL	CHN	COL
21	22	23	24	25	26	27	28	29	30
CIV	CUB	CZE	COD	DOM	ECU	EGY	ETH	FRA	DEU
31	32	33	34	35	36	37	38	39	40
GHA	GRC	GTM	GIN	HTI	IND	IDN	IRN	IRQ	ITA
41	42	43	44	45	46	47	48	49	50
JPN	JOR	KAZ	KEN	MDG	MWI	MYS	MLI	MEX	MAR
51	52	53	54	55	56	57	58	59	60
MOZ	MMR	NPL	NLD	NER	NGA	PAK	PER	PHL	POL
61	62	63	64	65	66	67	68	69	70
PRT	ROU	RUS	RWA	SAU	SEN	SOM	ZAF	KOR	SSD
71	72	73	74	75	76	77	78	79	80
ESP	LKA	SDN	SWE	SYR	CNG	THA	TUN	TUR	UGA
81	82	83	84	85	86	87	88	89	90
UKR	GBR	TZA	USA	UZB	VEN	VNM	YEM	ZMB	ZWE

**Table 2:** Weekly (from Week 15 to 51) COVID-19 cases (c) and deaths (d) for Afghanistan, which is indicated by 1, and Algeria, which is indicated by 2.

**Week**	**1c**	**2c**	**1d**	**2d**	**Week**	**1c**	**2c**	**1d**	**2d**
15	308	594	11	141	34	403	2,877	12	65
16	389	715	15	82	35	163	2,686	15	66
17	535	753	24	50	36	236	2,218	10	55
18	1,173	1,092	28	38	37	318	1,890	8	56
19	1,698	1,249	35	39	38	328	1,572	21	60
20	2,262	1,296	49	46	39	183	1,241	12	42
21	3,918	1,287	49	52	40	114	1,069	9	46
22	4,623	1,088	39	53	41	458	936	15	41
23	5,137	760	100	54	42	401	1,330	15	55
24	4,424	765	114	60	43	633	1,741	22	58
25	4,067	852	110	78	44	800	2,129	27	59
26	2,134	1,502	140	52	45	606	3,779	24	75
27	1,984	2,668	143	55	46	1,164	5,628	61	106
28	1,500	3,254	146	59	47	1,368	7,183	69	121
29	1,024	3,889	171	67	48	1,073	7,359	68	135
30	788	4,273	88	77	49	1,672	6,031	137	106
31	447	4,108	15	76	50	1,757	3,850	71	80
32	344	3,749	28	71	51	740	3,101	111	70
33	542	3,369	63	68					

**Table 3:** Weekly growth rates of cases and deaths for COVID-19 from Week 15 to 50 for Afghanistan (AFG) and Algeria (DZA).

**Week**	**15**	**16**	**17**	**18**	**19**	**20**
Weekly growth rate: AFG case	0.262	0.374	1.19	0.447	0.332	0.732
Weekly growth rate: DZA case	0.203	0.053	0.45	0.144	0.038	−0.007
Weekly growth rate: AFG death	0.013	0.023	0.007	0.006	0.008	0
Weekly growth rate: DZA death	−0.099	−0.045	−0.016	0.001	0.006	0.005
**Week**	21	22	23	24	25	26
Weekly growth rate: AFG case	0.18	0.111	−0.139	−0.081	−0.475	−0.07
Weekly growth rate: DZA case	−0.155	−0.301	0.007	0.114	0.762	0.776
Weekly growth rate: AFG death	−0.003	0.013	0.003	−0.001	0.007	0.001
Weekly growth rate: DZA death	0.001	0.001	0.008	0.023	−0.03	0.002
**Week**	27	28	29	30	31	32
Weekly growth rate: AFG case	−0.244	−0.317	−0.23	−0.432	−0.23	0.574
Weekly growth rate: DZA case	0.22	0.195	0.099	−0.039	−0.087	−0.101
Weekly growth rate: AFG death	0.002	0.017	−0.081	−0.093	0.029	0.101
Weekly growth rate: DZA death	0.001	0.002	0.003	0	−0.001	−0.001
**Week**	33	34	35	36	37	38
Weekly growth rate: AFG case	−0.256	−0.594	0.445	0.346	0.031	−0.441
Weekly growth rate: DZA case	−0.146	−0.066	−0.174	−0.148	−0.168	−0.21
Weekly growth rate: AFG death	−0.094	0.007	−0.030	−0.008	0.041	−0.027
Weekly growth rate: DZA death	−0.001	0	−0.004	0	0.002	−0.011
**Week**	39	40	41	42	43	44
Weekly growth rate: AFG case	−0.375	2.991	−0.124	0.577	0.263	−0.242
Weekly growth rate: DZA case	−0.138	−0.124	0.42	0.309	0.223	0.775
Weekly growth rate: AFG death	−0.016	0.052	0	0.017	0.008	−0.004
Weekly growth rate: DZA death	0.003	−0.005	0.015	0.002	0.001	0.008
**Week**	45	46	47	48	49	50
weekly growth rate: AFG case	0.919	0.175	−0.215	0.558	0.051	−0.578
weekly growth rate: DZA case	0.489	0.276	0.024	−0.18	−0.362	−0.194
weekly growth rate: AFG death	0.061	0.007	−0.001	0.064	−0.039	0.023
weekly growth rate: DZA death	0.008	0.003	0.002	−0.004	−0.004	−0.003

Based on this table and the featured frequencies (vectors), i.e., orthonormal basis 𝔹_*N*_ (or 𝔹_36_ in our case), one could then calculate (an example for AFG and DZA) their coefficients (or inner product) as shown in Table 4, in which the meaning of *b*_*j*_ is explained in section 2.1.

**Table 4:** Coefficients, or inner product, for 36 frequencies (or features) with respect to weekly growth rates of cases and deaths for Afghanistan (AFG) and Algeria (DZA).

**Frequency**	***b*_0_**	***b*_1_**	${\tilde{b}}_{1}$	***b*_2_**	${\tilde{b}}_{2}$	***b*_3_**
AFG cases	−0.276	0.162	1.269	−0.422	0.782	0.306
DZA cases	0.205	0.198	0.2	−0.4	0.285	0.144
AFG deaths	−0.043	0.006	0.043	0.027	−0.03	−0.003
DZA deaths	−0.021	−0.036	−0.032	−0.029	−0.022	−0.019
**Frequency**	${\tilde{b}}_{3}$	***b*_4_**	${\tilde{b}}_{4}$	***b*_5_**	${\tilde{b}}_{5}$	***b*_6_**
AFG cases	−0.707	1.259	−0.204	−0.588	1.255	−0.217
DZA cases	0.304	0.144	−0.07	0.234	−0.047	0.35
AFG deaths	0	0.07	−0.065	0.016	0.049	−0.021
DZA deaths	−0.014	−0.027	−0.031	−0.021	−0.022	−0.007
**Frequency**	${\tilde{b}}_{6}$	***b*_7_**	${\tilde{b}}_{7}$	***b*_8_**	${\tilde{b}}_{8}$	***b*_9_**
AFG cases	−0.438	−0.983	0.422	0.598	−0.116	1.057
DZA cases	0.882	−0.003	−0.568	0.027	−0.2	0.346
AFG deaths	0.005	0.006	−0.041	0.04	0.007	0.048
DZA deaths	−0.021	−0.032	−0.036	−0.015	−0.021	−0.021
**Frequency**	${\tilde{b}}_{9}$	***b*_10_**	${\tilde{b}}_{10}$	***b*_11_**	${\tilde{b}}_{11}$	***b*_12_**
AFG cases	0.696	0.033	−0.191	0.229	0.545	−1.269
DZA cases	−0.105	0.067	0.116	−0.022	0.297	0.075
AFG deaths	−0.043	−0.009	0.04	0.006	−0.011	−0.068
DZA deaths	−0.006	−0.003	−0.012	−0.02	−0.013	−0.014
**Frequency**	${\tilde{b}}_{12}$	***b*_13_**	${\tilde{b}}_{13}$	***b*_14_**	${\tilde{b}}_{14}$	***b*_15_**
AFG cases	0.513	0.513	−1.018	0.152	0.18	−0.607
DZA cases	−0.125	−0.066	−0.201	0.062	0.251	0.179
AFG deaths	0.035	0.075	−0.027	−0.075	0.006	0.042
DZA deaths	−0.006	0.01	0.002	−0.001	−0.008	−0.021
**Frequency**	${\tilde{b}}_{15}$	***b*_16_**	${\tilde{b}}_{16}$	***b*_17_**	${\tilde{b}}_{18}$	***b*_18_**
AFG cases	0.557	−0.778	0.038	−0.085	−0.786	−0.308
DZA cases	0.034	−0.292	−0.475	−0.73	0.41	−0.286
AFG deaths	0.039	−0.071	−0.001	0.017	0.007	0.001
DZA deaths	−0.013	−0.009	−0.007	−0.007	−0.007	−0.033

Now I rank the coefficients. A higher positive integer is assigned, if a coefficient is higher. The assignment for each country (here I present only AFG and DZA) is shown in Table 5. The distances of ranked vectors between different countries could then be calculated by Manhattan metric. The results are shown in Table 6. Based on these distance matrices, one could associate each country with its nearest neighbor(s) with respect to cases and deaths. The results are presented in Table 7. In the table, “Cty” stands for Country. Since the death rate for Country 14 is 0, the associated values are ignored when it is involved. Some countries might associate with more than one country.

**Table 5:** Ranking the coefficients calculated in Table 4 for Afghanistan (AFG) and Algeria (DZA).

**Frequency**	***b*_0_**	***b*_1_**	${\tilde{b}}_{1}$	***b*_2_**	** ${\tilde{b}}_{2}$ **	***b*_3_**	** ${\tilde{b}}_{3}$ **	***b*_4_**	** ${\tilde{b}}_{4}$ **	***b*_5_**	** ${\tilde{b}}_{5}$ **	***b*_6_**
AFG cases	12	21	36	10	32	24	6	35	14	8	34	13
DZA cases	27	25	26	4	30	23	32	22	11	28	13	34
AFG deaths	5	19	32	26	8	13	15	35	4	24	34	10
DZA deaths	15	1	5	7	10	18	21	8	6	11	9	29
**Frequency**	${\tilde{b}}_{6}$	***b*_7_**	${\tilde{b}}_{7}$	***b*_8_**	${\tilde{b}}_{8}$	***b*_9_**	${\tilde{b}}_{9}$	***b*_10_**	${\tilde{b}}_{10}$	***b*_11_**	${\tilde{b}}_{11}$	***b*_12_**
AFG cases	31	18	15	23	28	1	27	26	2	20	22	7
DZA cases	10	19	21	14	31	20	9	12	7	18	29	24
AFG deaths	6	12	30	18	11	3	27	36	9	1	21	31
DZA deaths	32	33	24	17	23	20	31	36	35	34	26	13
**Frequency**	${\tilde{b}}_{12}$	***b*_13_**	${\tilde{b}}_{13}$	***b*_14_**	${\tilde{b}}_{14}$	***b*_15_**	${\tilde{b}}_{15}$	***b*_16_**	${\tilde{b}}_{16}$	***b*_17_**	${\tilde{b}}_{18}$	***b*_18_**
AFG cases	29	5	19	17	4	11	9	3	25	30	16	33
DZA cases	17	5	3	1	35	6	36	15	2	16	8	33
AFG deaths	28	2	14	25	23	16	17	20	7	29	22	33
DZA deaths	22	25	27	30	28	3	12	4	2	19	16	14

*The higher the coefficients are, the higher the ranks are. The higher ranks also indicate the main features of weekly growth rates of COVID-19 in terms of the chosen 36 frequencies*.

**Table 6:** Manhattan distance (or $d(\vec{x},\vec{y}):\;=\sum_{i=1}^{n}|{\vec{x}}_{i}-{\vec{y}}_{i}|$) matrix, which is calculated from table for 90 countries with respect to COVID-19 cases (top block) and COVID-19 deaths (bottom block).

**Countries**	**1**	**2**	**3**	**4**	**⋯**	**87**	**88**	**89**	**90**
1	0	428	444	416	⋯	450	340	432	400
2	428	0	428	386	⋯	390	350	400	402
3	444	428	0	316	⋯	416	394	388	430
4	416	386	316	0	⋯	370	462	400	422
⋮	⋮	⋮	⋮	⋮	⋮	⋮	⋮	⋮	⋮
87	450	390	416	370	⋯	0	404	332	418
88	340	350	394	462	⋯	404	0	408	462
89	432	400	388	400	⋯	332	408	0	374
90	400	402	430	422	⋯	418	462	374	0
**Countries**	**1**	**2**	**3**	**4**	**⋯**	**87**	**88**	**89**	**90**
1	0	468	416	484	⋯	424	494	412	426
2	468	0	410	470	⋯	434	462	472	426
3	416	410	0	366	⋯	444	468	406	396
4	484	470	366	0	⋯	446	336	446	408
⋮	⋮	⋮	⋮	⋮	⋮	⋮	⋮	⋮	⋮
87	424	434	444	446	⋯	0	486	426	402
88	494	462	468	336	⋯	486	0	430	500
89	412	472	406	446	⋯	426	430	0	438
90	426	426	396	408	⋯	402	500	438	0

*This distance is derived directly from Table 5*.

**Table 7:** Minimal pairs, in term of Manhattan distances from Table 6, for COVID-19 cases and deaths for the 90 countries.

**Cty**	**1**	**2**	**3**	**4**	**5**	**6**	**7**	**8**	**9**	**10**	**11**	**12**
Cases	72	84	37	86	43	44	57	54	89	20	88	23
Deaths	14	19	78	9	9, 10, 27	15	37	82	4	25	17	8
Cty	13	14	15	16	17	18	19	20	21	22	23	24
Cases	53	28	24	8	70	10	77	10	39	41	8	27
Deaths	19	–	6	54	11	55	13	39	–	83	84	–
Cty	25	26	27	28	29	30	31	32	33	34	35	36
Cases	11	33,51	24	14	90	44,55	35	74	26	24	31	17,18
Deaths	10	53	5	35	76	54	72	41	86	–	28	11
Cty	37	38	39	40	41	42	43	44	45	46	47	48
Cases	3,4	74	57	60	43	35	5	90	66	90	85	67
Deaths	7	47	20	54	30,79	52	31	17	67	48	–	66
Cty	49	50	51	52	53	54	55	56	57	58	59	60
Cases	66	55	26	69	13	8	50	10	7	10	90	40
Deaths	70	80	16	42	26	30	89	17	11	80	76	62
Cty	61	62	63	64	65	66	67	68	69	70	71	72
Cases	40	60	70	69	57	7	48	88	64	17	8	1
Deaths	82	60	56	–	73	48	45	49	52	49	36	31
Cty	73	74	75	76	77	78	79	80	81	82	83	84
Cases	7	81	4	78	19	55,76	76	65	74	19	78	2
Deaths	65	15	89	29	78	77	30	58	62	8	22	13
Cty	85	86	87	88	89	90						
Cases	47	4	14	11,70	28	46						
Deaths	61	90	10	20	55	86						

## 4. Discussion

### 4.1. Comparison

Here I utilize another typical approach, namely: cosine similarity, to compare our method with others. Though the cosine similarity is highly frequently used in many fields, it focuses less on the some internal structures. For example, if $\vec{p}=\left(5,4\right),\vec{q}=\left(-4,5\right),\vec{r}=\left(1,-\frac{5}{4}\right)$. Then $cos\left(\vec{p},\vec{q}\right)=cos\left(\vec{p},\vec{r}\right)=0$. But, with our ranked Manhattan metric (or *d*) $d\left(\vec{p},\vec{q}\right)=2$ and $d\left(\vec{p},\vec{r}\right)=0$. Moreover, when the coefficients are ranked, they tend to reduce the noise of the data—in particular, the cases and deaths are affected by many factors. The results of the cosine similarities for the 90 countries (except the for country 14, which is ignored for the part of deaths, due to its death cases are being zero). The results are presented in Table 8. Again, by linking each country to its neighbor which has the maximal cosine similarities, one has Table 9.

**Table 8:** Typical cosine similarities of COVID-19 cases (top block) and deaths (bottom block) for 90 countries (Cty).

**Cty**	**1**	**2**	**3**	**4**	**⋯**	**87**	**88**	**89**	**90**
1	1	0.032	0.099	0.112	⋯	−0.081	0.3	0.014	0.082
2	0.032	1	0.134	0.089	⋯	0.141	0.168	0.228	0.024
3	0.099	0.134	1	0.503	⋯	0.05	0.202	0.146	-0.057
4	0.112	0.089	0.503	1	⋯	0.284	0.046	0.26	0.204
⋮	⋮	⋮	⋮	⋮	⋮	⋮	⋮	⋮	⋮
87	-0.081	0.141	0.05	0.284	⋯	1	0.079	0.342	0.061
88	0.3	0.168	0.202	0.046	⋯	0.079	1	0.087	0
89	0.014	0.228	0.146	0.26	⋯	0.342	0.087	1	0.176
90	0.082	0.024	−0.057	0.204	⋯	0.061	0	0.176	1
**Cty**	**1**	**2**	**3**	**4**	**⋯**	**87**	**88**	**89**	**90**
1	1	−0.078	−0.009	−0.173	⋯	0.022	−0.073	0.134	−0.072
2	−0.078	1	0.085	0.097	⋯	0.005	−0.07	−0.043	0.696
3	−0.009	0.085	1	0.225	⋯	−0.012	0.039	0.005	0.011
4	−0.173	0.097	0.225	1	⋯	−0.001	0.245	−0.079	0.243
⋮	⋮	⋮	⋮	⋮	⋮	⋮	⋮	⋮	⋮
87	0.022	0.005	−0.012	−0.001	⋯	1	−0.033	−0.038	0.051
88	−0.073	−0.07	0.039	0.245	⋯	−0.033	1	−0.034	−0.081
89	0.134	−0.043	0.005	−0.079	⋯	−0.038	−0.034	1	−0.073
90	−0.072	0.696	0.011	0.243	⋯	0.051	−0.081	−0.073	1

**Table 9:** Maximal pairs, in terms of typical similarities, of COVID-19 cases and deaths for the 90 countries (Cty).

**Cty**	**1**	**2**	**3**	**4**	**5**	**6**	**7**	**8**	**9**	**10**	**11**	**12**
Cases	72	84	37	86	41	61	83	54	31	68	68	23
Deaths	58	86	78	9	10	13	15	82	4	87	56	8
Cty	13	14	15	16	17	18	19	20	21	22	23	24
Cases	90	28	57	82	70	11	77	10	39	32	8	27
Deaths	19	–	19	51	44	55	13	39	12	83	83	57
Cty	25	26	27	28	29	30	31	32	33	34	35	36
Cases	68	13	24	14	71	40	35	22	51	24	31	11
Deaths	86	53	9	35	30	54	72	60	35	7	28	11
Cty	37	38	39	40	41	42	43	44	45	46	47	48
Cases	4	6	20	30	5	35	5	90	66	90	82	73
Deaths	7	23	20	82	30	52	31	17	67	90	10	66
Cty	49	50	51	52	53	54	55	56	57	58	59	60
Cases	73	81	33	81	7	8	50	7	73	10	90	40
Deaths	11	86	16	42	26	30	18	11	63	80	76	62
Cty	61	62	63	64	65	66	67	68	69	70	71	72
Cases	40	60	7	69	7	56	48	10	64	88	8	1
Deaths	82	76	56	21	73	48	45	57	52	49	76	86
Cty	73	74	75	76	77	78	79	80	81	82	83	84
Cases	7	60	33	78	19	76	76	9	63	54	7	2
Deaths	88	82	19	29	78	77	30	58	34	8	22	19
Cty	85	86	87	88	89	90						
Cases	78	4	28	70	36	46						
Deaths	53	90	10	17	28	86						

### 4.2. Optimal Pairings

In this section, I list and compare the optimal minimal and maximal pairs from Tables 7, 9. The results are shown in Table 10. I could apply Jaccard Index $J\left(A,B\right)=\frac{\left|A\cap B\right|}{\left|A\cup B\right|}$ to analyze their relation, where *A, B* are sets.

**Table 10:** Optimal pairings for the countries with respect to cases and deaths in terms of minimal and maximal values.

			**Cases-min**			
{1, 72}	{2, 84}	{3, 37}	{4, 86}	{5, 43}	{7, 57}	{8, 54}
{10, 20}	{11, 88}	{13, 53}	{14, 28}	{17, 70}	{19, 77}	{24, 27}
{26, 33}	{31, 35}	{40, 60}	{46, 90}	{47, 85}	{48, 67}	{50, 55}
{26, 51}	{64, 69}	{74, 81}	{76, 78}			
			**Deaths-min**			
{4, 9}	{5, 27}	{6, 15}	{7, 37}	{8, 82}	{10, 25}	{11, 17}
{13, 19}	{20, 39}	{22, 83}	{26, 53}	{28, 35}	{29, 76}	{30, 54}
{31, 72}	{42, 52}	{45, 67}	{48, 66}	{49, 70}	{55, 89}	{58, 80}
{60, 62}	{65, 73}	{77, 78}	{86, 90}			
			**Cases-max**			
{1, 72}	{2, 84}	{4, 86}	{5, 41}	{7, 83}	{8, 54}	{10, 68}
{14, 28}	{19, 77}	{22, 32}	{24, 27}	{30, 40}	{31, 35}	{33, 51}
{46, 90}	{64, 69}	{70, 88}	{76, 78}			
			**Deaths-max**			
{4, 9}	{8, 82}	{10, 87}	{11, 56}	{13, 19}	{16, 51}	{17, 44}
{18, 55}	{20, 39}	{22, 83}	{26, 53}	{28, 35}	{30, 54}	{42, 52}
{45, 67}	{46, 90}	{48, 66}	{58, 80}	{77, 78}	{86, 90}	

## 5. Conclusion and Future Work

Based on our devised orthonormal basis, which is motivated by Fourier analysis, I perform spectral analysis on 90 representative 90 countries. The main purpose for such an analysis is to identify the patterns of evolution of COVID-19 across the globe. To this end, the coefficients which measure the relation between the growth rate of COVID-19 country and the given features in the spectrum are utilized. Then I rank the coefficients to reveal their internal structures and then apply the Manhattan metric to compute the distances between countries. This constructed distance matrix would reveal the relations between the countries regarding the evolution of COVID-19 cases and deaths. In addition, I also identify the nearest neighbor with respect to minimal distance via the distance matrix. By the end, I compare our mechanism with the usual cosine similarity analysis. The result shows these two approaches yield quite different results - this indicates our approach provides another aspect to look into the evolution of COVID-19. The comparison also reveals some points: first of all, the evolutive pattern for cases and deaths are very different—which is concluded from Table 11; secondly, regardless of the cases or the deaths, our method and the typical one are highly related to each other; and thirdly, the relation between the paired countries—no matter which approach one adopts—is not random, since the ratios of pairs formed are very high. This indicates our research provides some insightful structure of the evolution of COVID-19 between countries. However, some of the results about causal relations in this study might not comply with other researches (10). This is reasonable, since the approach I adopt focuses more on feature detection, not solely on causal relation finding. For the future research, one could look into the pairs to identify the fundamental factors that contribute to such correlated patterns between countries. Furthermore, one could also delve into the shift of phrases of the frequencies by lifting the constraint on weekly growth rates. This might yield an even more dynamical pictures of the evolutions.

**Table 11:** Jaccard index (or $J\left(A,B\right)=\frac{\left|A\cap B\right|}{\left|A\cup B\right|}$) for minimal and maximal pairings.

	**Cases-min**	**Deaths-min**	**Cases-max**	**Deaths-max**
Cases-min	1	0	$\frac{11}{32}$	$\frac{1}{44}$
Deaths-min	0	1	0	$\frac{14}{31}$
Cases-max	$\frac{11}{32}$	0	1	$\frac{1}{37}$
Deaths-max	$\frac{1}{44}$	$\frac{14}{31}$	$\frac{1}{37}$	1

## Data Availability Statement

The original contributions presented in the study are included in the article/supplementary material, further inquiries can be directed to the corresponding author/s.

## Author Contributions

The author confirms being the sole contributor of this work and has approved it for publication.

## Funding

This work was supported by the Humanities and Social Science Research Planning Fund Project under the Ministry of Education of China (No. 20XJAGAT001).

## Conflict of Interest

The author declares that the research was conducted in the absence of any commercial or financial relationships that could be construed as a potential conflict of interest.

## Publisher's Note

All claims expressed in this article are solely those of the authors and do not necessarily represent those of their affiliated organizations, or those of the publisher, the editors and the reviewers. Any product that may be evaluated in this article, or claim that may be made by its manufacturer, is not guaranteed or endorsed by the publisher.
